# Superellipse Equation Describing the Geometries of *Abies alba* Tree Rings

**DOI:** 10.3390/plants13243487

**Published:** 2024-12-13

**Authors:** Weiwei Huang, Kehang Ma, Jiaxin Tan, Meixiang Wei, Yunjie Lu

**Affiliations:** 1 Co-Innovation Center of Sustainable Forestry in Southern China, College of Forestry and Grassland, Nanjing Forestry University, Nanjing 210037, China; tanjiaxin@njfu.edu.cn (J.T.); weimeixiang@njfu.edu.cn (M.W.); yunjielu@njfu.edu.cn (Y.L.); 2 Department of Geosciences and Natural Resource Management, The University of Copenhagen, 1958 Frederiksberg C, Denmark; 3 College of Ecology and Environment, Nanjing Forestry University, Nanjing 210037, China

**Keywords:** tree-ring shape, Lamé curve, bilateral symmetry

## Abstract

Our previous study using 41 tree rings of one *Abies alba* Mill. disc indicated that the superellipse equation can accurately fit its tree-ring shape. This study further used the superellipse equation (${\left|\frac{x}{a}\right|}^{n}+{\left|\frac{y}{\beta }\right|}^{n}=1$ ) to model the geometries of 1090 tree rings of *A. alba* discs collected from five sites in Denmark. The adjusted root-mean-square-error (RMSE_adj_) was calculated to assess the goodness of fit between observed and predicted tree-ring boundaries. The results showed that RMSE_adj_ ranged between 0.0038 and 0.0591, with a mean value of 0.0141. This verified that the superellipse equation sufficiently describes the *A. alba* tree-ring shape. In the polar coordinate system, the superellipse equation can be expressed as $r={a({\left|cos\varphi \right|}^{n}+{\left|sin\varphi /k\right|}^{n})}^{-1/n}$. Where r and φ are the polar radius and polar angle, respectively. k=β/a, where a and β are the major and minor semi-axes of the superellipse. The mean value of *k* was 0.95, 94% of tree rings had *k*-values between 0.90 and 1.00, and only 67 tree rings had *k*-values between 0.71 and 0.90. *n*-value ranged from 1.62 to 2.81, with an average value of 2.04. 59% of the tree rings had *n*-values between 1.90 and 2.10, and 62% showed *n*-values greater than 2.0. This means that most tree rings are a hyperellipse approached to an ellipse. Sites with different soil moisture conditions influenced the size but not the shape of tree rings. This study verified that the tree-ring shape of *A. alba* tends to be bilaterally symmetric and hyperellipse approached ellipse. Its variation was reflected more in inter-annual differences in *k*- and *n*-values.

## 1. Introduction

The annual rings on the trunk of a tree are formed by new cells produced in the meristematic tissues that lie beneath the secondary phloem (i.e., the living bark). For temperate tree species, one annual ring usually consists of lighter-colored, less dense earlywood and darker-colored, denser latewood [1]. Earlywood forms at the beginning of the growing season, with cells proliferating and at a low density when the tree emerges from dormancy. Later in the growing season, when hot and dry conditions become more prevalent, cell growth slows, and wood density increases, forming latewood [2,3]. Tree rings can gauge information about precipitation, temperature, fire, and other data about that year and are sources of historical details in nature that record a tree’s growth trends [4]. The patterns of wide or narrow annual rings record inter-annual climate variations in the growth of trees [5,6,7,8,9]. The inter-annual variations in tree-ring widths and wood anatomy features have been used by ecologists to understand the relationships between tree growth and climate change [7,10,11,12] and used by geologists to construct paleoclimate [13,14], streamflow and hydrological characteristics [15,16], and forest fire history [17]. Archaeologists use tree-ring patterns on timbers to date the construction of some world-famous buildings and other wooden structures (e.g., musical instruments, ships, etc.) [18,19,20,21].

Dendrochronologists usually consider the shape of a tree ring to be close to a concentric circle [22,23]. However, in nature, most tree-ring shapes are not a circle but closer to an ellipse or even a distortion of an ellipse. This is because trees are constantly affected by the ever-changing natural environment as they grow. Since annual tree rings are formed by cambium cells whose formation and differentiation are influenced by genetic, environmental, and geographic factors, such inter-annual variations can act simultaneously on the tree-ring shapes [7,10,11,24,25,26]. For instance, reaction wood is formed under long-term mechanical loading, such as a slope and strong winds. Changing the shape of the tree disc to an ellipse-related ring shape helps restore the main stem’s vertical growth, orient the crown toward the sunlight, and hold the branches in position. Variations in monthly, seasonal, and annual precipitation and temperature alter the differentiation and formation of cambium cells, which in turn influence the tree-ring width.

**Figure 1:**
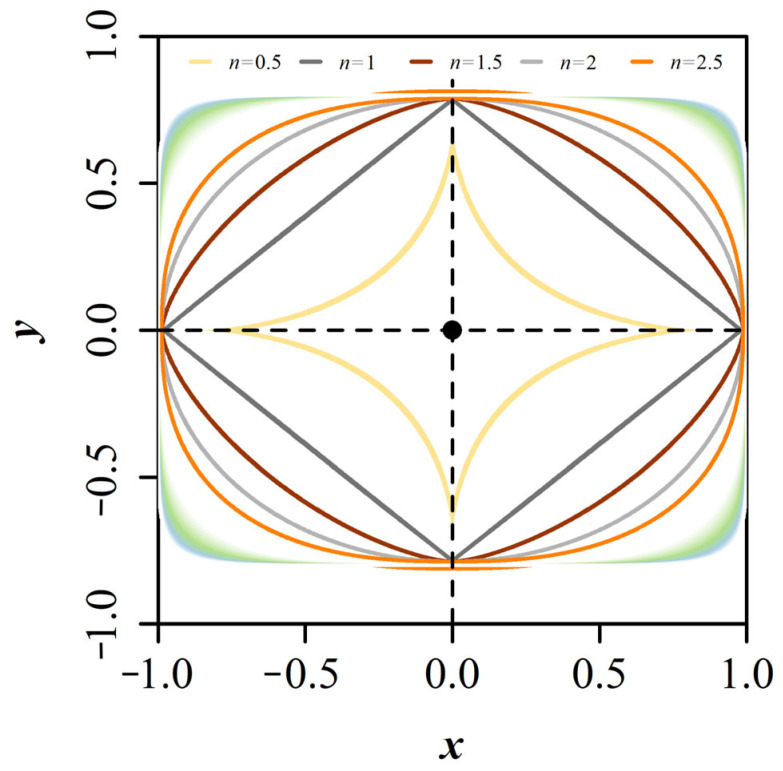
Simulated superellipses. *α* = 1, *β* = 0.80, and *n* ranges from 0.01 to 10 in increments of 0.01. Isolines for five simulated superellipses with *n* = 0.5, 1, 1.5, 2, and 2.5 were shown.

What kind of geometric function could be used to describe the actual tree-ring shape? Since trees are constantly affected by an ever-changing environment, circular equations are no longer sufficient to fit the shape of the tree-ring accurately. Skatter and Høibø [27] compared circular, elliptic, and Fourier’s transformations to fit *Picea abies* (L.) Karst., and *Pinus sylvestris* L. disc shape and found that the elliptic model was the best. Further, Saint-André and Leban [28] analyzed 453 *P. abies* tree discs with 9500 annual rings and showed that the compound ellipse model proposed by Bindzi et al. [29] was slightly better than the single ellipse model. However, this model is more complex, and the root-mean-square-error (RMSE) of the single ellipse is about 0.4 cm, which is 3.2% of the average radius so good results can be obtained. Leaning, landslide activity, or wind-driven tensions will shift the center of annual ring growth. Saint-André and Leban [28] showed that tree rings were rather circular near the pith and top of the trunk and rather elliptical near the bark and bottom of the stem. Visser et al. [23] introduced the quadro-ellipse model to simulate more complex eccentric tree-ring shapes, where both the eccentricity and the growth center may evolve over time. In 1818, Lamé [30] introduced a generalized version of the ellipse equation, now known as Lamé curves or superellipses (Figure 1). The superellipse equation generates Euclidean geometries by introducing a shape parameter, i.e., *n*, into the elliptic equation. For *n* = 2, the superellipse equation gives a typical ellipse; *n* < 2 produces a hypoellipse; *n* > 2 makes a hyperellipse. The superellipse equation has been widely applied to fit a variety of natural shapes, e.g., cross-sectional shape of square bamboo culm [31], coniferous tree-ring shape [26,32], fruit 2D shape [33], leaf stomatal and leaf shape [34,35], seed shape [36], etc. In our previous study, the geometries of tree-ring shapes for six coniferous species were modeled by a superellipse equation with adjusted root-mean-square-error (RMSE_adj_) for the 255 rings ranging from 0.0026 to 0.0394 with a mean of 0.0132 [26]. This is even though many tree stems are eccentric due to the effects of a changing environment. Most of the studied tree-ring shapes of the six conifer species tended to be bilaterally symmetrical, meaning that most tree rings had their geometric centers, even though they were not in the pith [26]. Since our previous study examined the annual rings of just one disc per tree species, the “standard” *n*-value of the superellipse equation for coniferous species requires a large number of samples to be validated.

*Abies alba* Mill. is a long-lived, evergreen tree and the most giant tree of the native Abies in Europe. This species has a deep and dense taproot system, readily biodegradable needle litter, and shade tolerance, making it ecologically and silviculturally crucial for establishing and managing site-appropriate mixed forests with stability. *A. alba* wood is strong, long-fibered, lightweight, fine-grained, uniform, light-colored, and free of primary resin tubes. It is mainly used as construction timber, furniture, plywood, and pulpwood. A number of ring shapes have been suggested in the literature, namely circles, ellipses, and quadro-ellipse. In our previous study, we proposed two more ring shapes, namely the superellipse and the Gielis equation and the superellipse equation is sufficient to describe the tree-ring boundary and estimate the tree-ring area of 41 tree rings of one *A. alba* disc [26]. In production practice, suitable equations to calculate the area of a trunk can reduce the overestimation or underestimation of the basal area.

This study is based on a large dataset with the objectives of (i) testing the validity of the superellipse equation in describing the shape of *A. alba* tree rings; (ii) investigating whether the *A. alba* tree-ring shapes are close to the ellipse, hyperellipse or hypoellipse, as indicated by the parameter *n* of the superellipse equation; (iii) assessing the coefficients of variation of *n*-value and the ratio of major and minor radius among tree rings within the tree disc, and exploring their site difference.

## 2. Results

**Figure 2:**
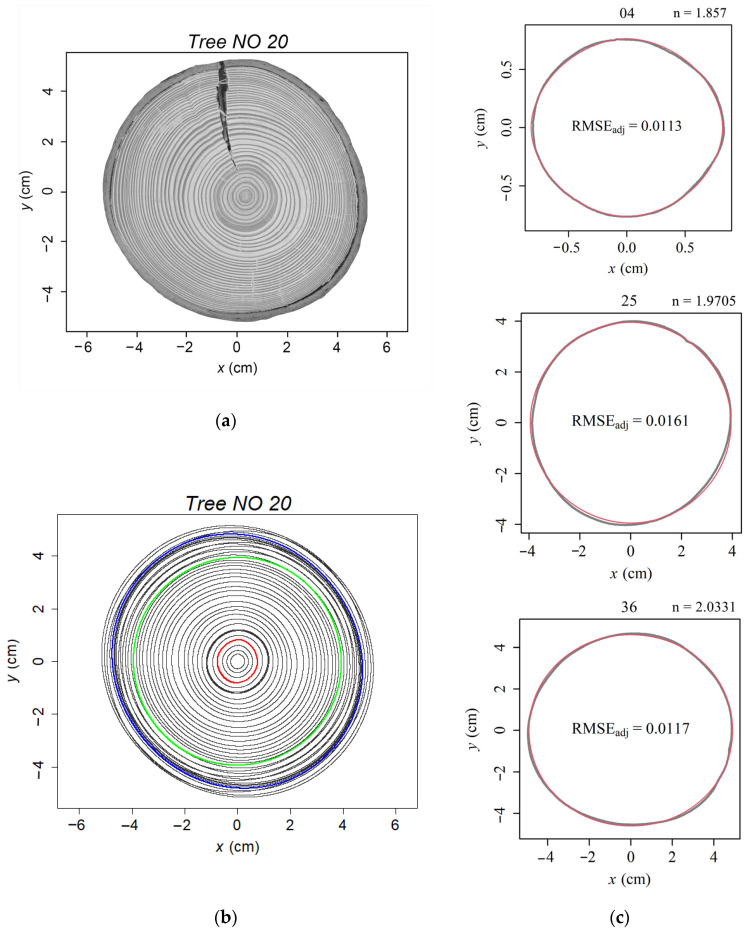
Images of the studied tree disc (NO 20) with the smallest coefficient of variation of parameter *n*. (**a**) Scanned image of studied tree-ring disc of tree 20; (**b**) Fitted results of superellipse equation to tree 20. The point with coordinate 0 (*x* = 0, *y* = 0) is the geometric center of each tree ring, not the pith; (**c**) The observed shapes (grey curves) and the fitted shapes (red curves) of the ring boundaries of three tree rings using the superellipse equation. The number on top of each tree-ring figure was the tree-ring number from pith to bark in the transverse section of tree 20. RMSE_adj_ is the adjusted root-mean-square-error indicating the accuracy of the superellipse model fitting.

**Figure 3:**
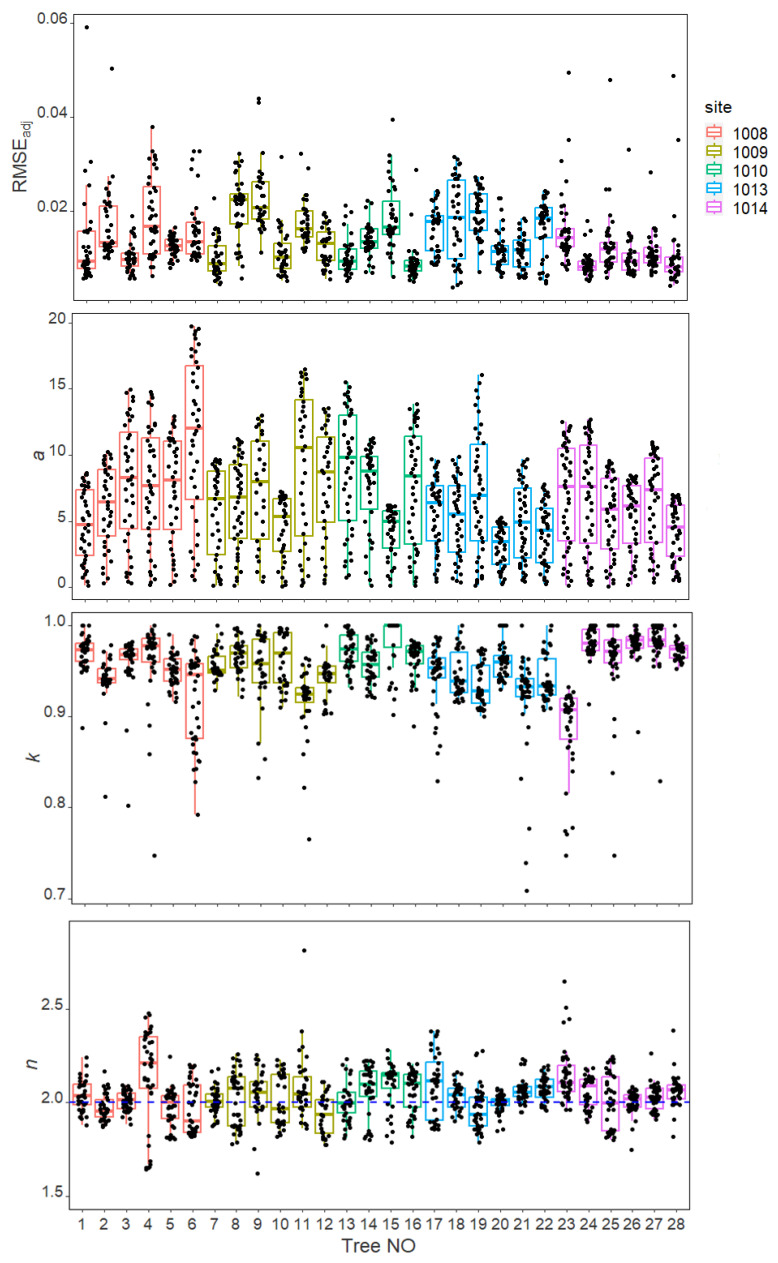
The estimates of the adjusted root-mean-square-error (RMSE_adj_), superellipse equation parameters *a*, *k*, and *n* for 1090 tree rings of 28 Silver fir trees. The horizontal solid line indicates the median and the whiskers extend to 1.5 times the interquartile range from the top and bottom of the box. The blue dashed horizontal line shows the *n* value of 2.

The predicted tree-ring curves of *A. alba* based on the superellipse equation closely matched the observed tree-ring boundaries (Figure 2c). The average RMSE_adj_ of each tree disc is smaller than 0.023, which means that the average absolute deviation between the actual and the predicted tree-ring radius did not exceed 2.3% of the radius of a hypothetical circle with an area equal to the ellipse of the tree-ring (Figure 3). Each studied tree disc’s mean estimated *a*-value (the major semi-axes) ranged from 3.13 to 11.32 cm (Figure 3). The mean estimated *k*-value (k=β/a, the β is the minor semi-axes) ranged from 0.89 to 0.98 with a mean value of 0.95 across all the 1090 studied tree rings (Figure 3 and Table 1). As the value of *k* approaches 1, the shape of the tree ring becomes more circular. The mean value of *n* across 1090 tree rings is 2.04, and the mean *n*-value of each studied tree disc ranged from 1.93 to 2.15 (Figure 3 and Table 1). The estimated *n*-value indicates that the studied *A. alba* tree-ring shapes are close to typical ellipses.

**Figure 4:**
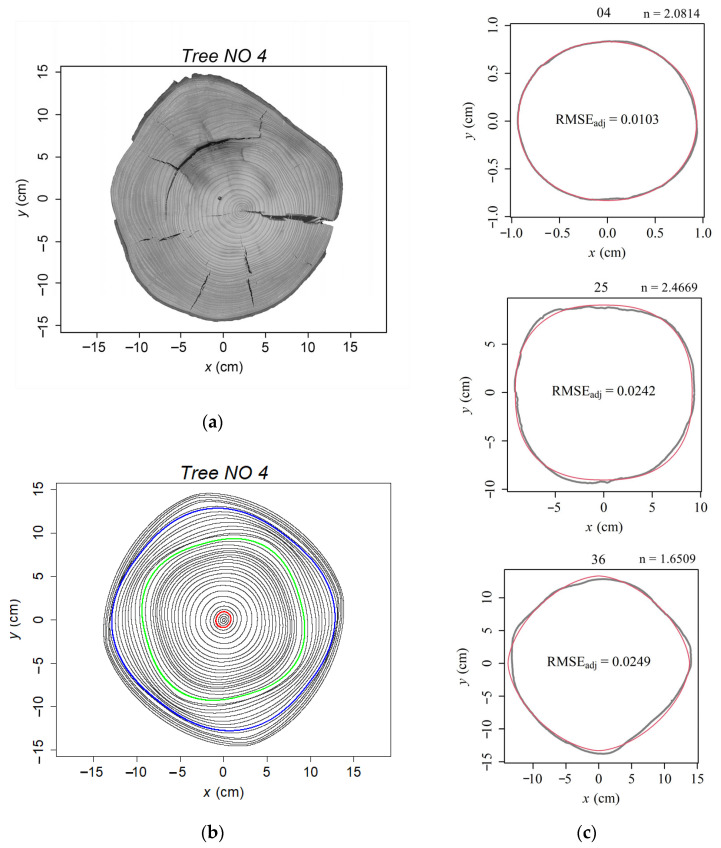
Images of the studied tree disc (NO 4) with the largest coefficient of variation of parameter *n*. (**a**) Scanned image of studied tree-ring disc of tree 4; (**b**) Fitted results of superellipse equation to tree 4. The point with coordinate 0 (*x* = 0, *y* = 0) is the geometric center of each tree ring, not the pith; (**c**) The observed shapes (grey curves) and the fitted shapes (red curves) of the ring boundaries of three tree rings using the superellipse equation. The number on top of each tree-ring figure was the tree-ring number from pith to bark in the transverse section of tree 4. RMSE_adj_ is the adjusted root-mean-square-error indicating the accuracy of the superellipse model fitting.

There were non-significant differences in the means and coefficients of variation (CV) of *n*- and *k*-value between sites (Table 2). Compare the *k*- and *n*-values among tree rings within each studied tree disc. Among the 28 studied tree discs, the coefficients of variation of *k*-value ranged from 0.98 to 6.29 with an average value of 3.00, and the coefficients of variation of *n*-value ranged from 2.49 to 12.03 with a mean value of 5.35 (Table 1). Tree 20 had the smallest *n*-CV with a value of 2.488, and Tree 4 had the largest *n*-CV with 12.026. Their scanned stem disc image and fitted tree-ring curves by superellipse equation were shown in Figure 2 and Figure 4, respectively. The *n*-value of the 40 tree rings of Tree 20 ranged from 1.85 to 2.08, and that of Tree 4 ranged from 1.64 to 2.47. The value of *n* approaches 2, the shape of a tree-ring boundary close to a typical ellipse (or a circle when k=1). For *n* > 2, it produces hyperellipse (Figure 4c); For *n* < 2, the superellipse equation produces hypoellipse (Figure 4c).

## 3. Discussion

Based on 1090 tree rings of *A. alba* tree-ring discs, this study showed that the superellipse equation can precisely fit the *A. alba* tree-ring shape. Thanks to the common garden experiment, the trees we studied were of the same age, but the size of the disk varied. The different sizes of the tree disks did not affect the fitting of the superellipse equation to the shape of the tree rings. The adjusted root-mean-square-error (RMSE_adj_) of 1090 studied tree rings ranged from 0.0038 to 0.0591 with an average value of 0.0141. Thus, the superellipse equation can be used to precisely describe the tree-ring shape of *A. alba*, which tends to be bilaterally symmetrical. The value of parameter *n* of 1090 studied tree rings ranged from 1.62 to 2.81, with an average value of 2.04. In addition, the *n*-value of 59% of studied tree rings ranged between 1.90 and 2.10. The rest of the tree-ring shapes deviate significantly from the standard ellipse. Theoretically, if the tree-ring shape followed the ellipse/circle equation, the *n*-value would equal or close to 2. The tree-ring shapes were hyperellipse (*n* > 2) in some years and changed to hypoellipse (*n* < 2) in other years. The inter-annual variations were different among different discs. In addition, 62% of tree rings had *n*-value higher than 2, which means most of the tree-ring shapes were hyperellipse, rather than hypoellipse. The variation of the ratio of minor to major semi-axis (*k*-value) ranged from 0.71 to 1.00, with an average value of 0.95. Specifically, only 67 tree rings of the studied 1090 tree rings had a *k*-value smaller than 0.90, meaning the variation of the ratio of minor to major semi-axis (*k*-value) of most tree rings was within the range between 0.90 and 1.00. The results of the current study obtained similar ellipticity rates to previous studies on conifers (i.e., *Pseudotsuga menziesii* (Mirb.) Franco, *Pinus pinaster* Ait., *P. abies*, *Abies grandis* Lindl., *Larix kaempferi* (Lamb.) Carr., *Picea sitchensis* (Bong.) Carr.) with ratio of minor to major semi-axis ranging from 0.91 to 0.98 [26,28,37,38]. Therefore, based on the *k*- and *n*-value fluctuation, the tree-ring shapes of *A. alba* can be better described by the superellipse equation rather than a circle or a pure ellipse equation [26,32].

Environmental differences between sites (topography, soil structure and fertility, stand structure, moisture, temperature, wind, etc.) can influence the formation and differentiation of xylem cells, which in turn affects annual increment growth [5,39,40]. Trends of annual increments of *P. abies* trees in relation to monthly precipitation and temperature were similar to that of *A. alba* grown in Denmark [5]. Park and Spiecker [41] analyzed the wood xylem characterize of *P. abies* trees from two sites in southwestern Germany. The results showed that trees from warmer, drier regions typically have more latewood cells and thicker cell walls, while ones from cooler, wetter regions have larger earlywood cells and thinner cell walls. These differences are attributed to the hydraulic adaptation mechanisms of trees to site-specific conditions. Cabon et al. [42] analyzed 1800 regional ring width chronologies of a large number of tree species from 31 North American and European sites, including *A. alba*, and found that the regional ring width was positively associated with site temperature and water availability. In our previous study, we analyzed the same *A. alba* tree discs found that as soil moisture in the site increased, the average annual increment of the sampled trees increased [5]. This means that the soil moisture conditions in the sample site influenced the size of the tree ring, but does it affect its shape? However, the analysis of variance (ANOVA) results showed that the mean value and coefficients of variation (CV) of parameters *n* and *k* were non-significant among the studied sites. The variation in the shape of tree rings exhibited through the values of parameters *n* and *k* of the superellipse equation was more reflected in the inter-annual differences, with *k*-CV and *n*-CV ranging between 0.983–6.286 and 2.488–12.026, respectively, among 28 studied trees. For instance, Tree 20 had the smallest CV of *n*-value (*n*-CV = 2.488; Figure 2) and mean *n*-value of 1.996. In Figure 2, most of the tree-ring shapes approached an ellipse. Tree 4 had the most considerable CV of *n*-value (*n*-CV = 12.026; Figure 4), and the mean *n*-value is 2.147. With inter-annual variation tree-ring shape fluctuates continuously from ellipse to hyperellipse to hypoellipse. In some years, even close to a rectangle-like with four rounded corners or a rhombus with four outwards curved corners. As the age of the tree changes, the four corners of the tree rings rotate, and this phenomenon is a cross-sectional representation of the Spiral grain of the trunk. Spiral grain is the difference between the longitudinal direction of the tracheids and the longitudinal axis of the tree trunk of a conifer, expressed as an angle between these two directions [43]. Many reasons have been considered to cause spiral grain [44]. For instance, when the distribution of the roots and canopy is uneven, the tracheids follow a spiral path, which could help transport water and nutrients to the crown. Physical factors, e.g., strong wind, an asymmetrical crown, and gravity, can also result in spiral grain [45]. Persistent, intense wind pressure can cause asymmetrical growth on both sides of the tree, resulting in compression wood on the leeward side [46,47]. However, the hypoellipse- or hyperellipse-like tree-ring shape of tree 4 was bilaterally symmetrical. We hypothesize that this change in angle and tree-ring shape may be due to the change in wind direction between years and within years. The spiral grain of the outmost part of the stem is proposed as a strategy to improve the strength of trees against the winds [48]. In future research, it would be interesting to incorporate historical data on wind force and direction using materials from common garden experiments to compare spiral grain characteristics of different species.

Based on 1090 tree rings, we consolidated our earlier preliminary findings that the tree-ring shape of *A. alba* tends to be bilaterally symmetrical, and the superellipse equation can describe the tree-ring shapes of *A. alba* sufficiently. In production practice, a circle equation was usually used to calculate the area of a trunk, which may often overestimate or underestimate the basal area. A pure ellipse equation that includes the minor and major axes will be more precise than a circle equation. However, the circle/ellipse equation does not consider that the changing environment continuously affects tree-ring growth. This is evidenced by the results of this study demonstrating changing *n*-values between years and inter-annual variation tree-ring shape fluctuated continuously from hypoellipse to ellipse to hyperellipse, and vice versa. Although the superellipse equation can better describe tree-ring shape than the circle/ellipse equation, for standing trees in forests, we more often measure annual increments by taking tree cores or measuring diameter at breast height (DBH) to calculate standing tree stock. Large numbers of eccentric tree rings are present in the field, and given that the geometric centers of tree-ring shapes change with tree age [23,28], the superellipse equation is more practical for simulating the basal area of tree discs, rather than tree cores.

## 4. Materials and Methods

### 4.1. Sample Collection

**Figure 5:**
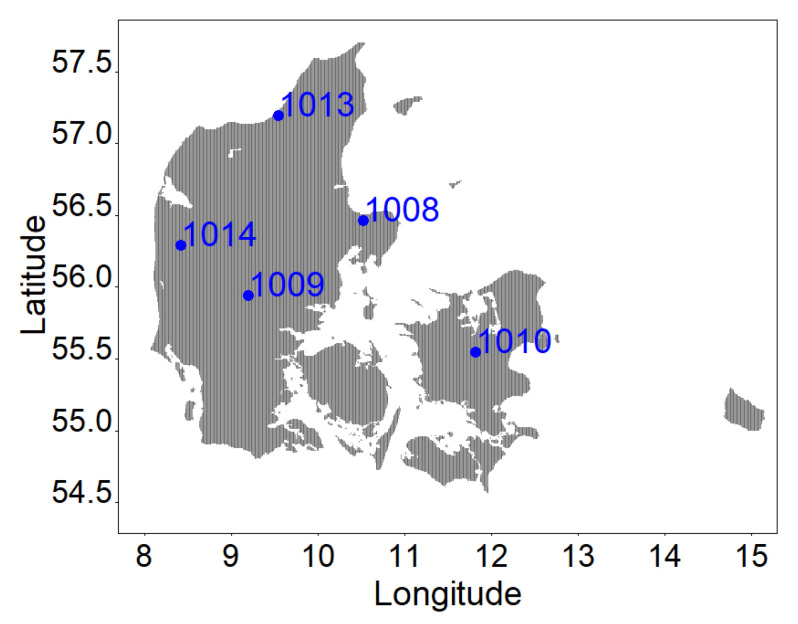
Map of the studies sites located in Denmark.

In the winter of 1964–1965, a nationwide Danish common garden experiment was established, including 13 field trails with ten conifer and two broadleaved species. The field trials consisted of 0.17–0.31 ha monospecific plots, randomly distributed in each trial. At all the sites, each species has the same Danish landrace or provenance [49]. Trees were planted at 2–4 years old, with a spacing of 1.3 × 1.3 m for conifers and 1.3 × 0.65 m for broadleaf trees. In the winter of 2012, trees were regularly thinned, and stem discs at breast height (1.3 m) of *A. alba* (silver fir) trees among the thinning trees from five sites were collected and used in the current study (Figure 5). In total, 1090 tree rings of 28 silver fir trees were analyzed (Table 1). The breast height diameter (DBH) of the 28 trees studied ranged from 11.40 cm to 40.00 cm with an average value of 23.49 cm.

### 4.2. Sample Preparation and Data Acquisition

To avoid decay, the 28 studied stem discs were oven-dried, and then sandpaper was used to polish the disc surface. A flatbed distortion-free scanner collected the digital images of the disc surfaces (Epson (Suwa, Japan), Expression 11000XL, Figure 2a and Figure 4a). Adobe Photoshop 2023 (version 24.0.1; Adobe Systems Incorporated, San Jose, CA, USA) was used to manually redraw the outer boundary of each tree ring (hereafter simply “boundary”). There were, in total, 1090 rings (232, 209, 160, 241, and 248 for sites 1008, 1009, 1010, 1013, and 1014, respectively). The digital images were converted into black and white BMP images with interior space inside redrawn tree-ring boundaries filled with black color using Adobe Photoshop 2023 (version 24.0.1; Adobe Systems Inc., San Jose, CA, USA). The boundary coordinates of each tree ring were extracted using a MATLAB procedure (version ≥ 2009a; MathWorks, Natick, MA, USA) developed by Shi et al. [50]. The digitization of the 2D profile of each tree ring was performed using the ‘adjdata’ function within the ‘biogeom’ package (version 1.3.5) based on R software [51]. The number of approximately equidistant data points forming each tree-ring perimeter was fixed at 600.

### 4.3. Parametric Fitting and Data Analysis

Lamé [30] introduced a generalized version of the ellipse equation to describe crystal shapes, and the superellipse equation is defined analytically by the formula:

(1) $${\left|\frac{x}{a}\right|}^{n}+{\left|\frac{y}{\beta }\right|}^{n}=1$$

where *x* and *y* are the horizontal and vertical coordinates of a point on the superellipse curve, a and β are the major and minor semi-axes of the superellipse, and *n* is an arbitrary positive actual number. For *n* < 2, the superellipse equation produces hypoellipse; For *n* > 2, it produces hyperellipse; For *n* = 2, it gives a typical ellipse (or a circle when a = β, see Figure 1 for visual representation with values of *n*, ranging from 0.5 to 2.5).

In the polar coordinate system, the superellipse equation can be expressed as:

(2) $$r={a({\left|cos\varphi \right|}^{n}+{\left|sin\varphi /k\right|}^{n})}^{-1/n}$$

where r and φ are the polar radius and polar angle, respectively. The a, k, and *n* are the three shape parameters, and k=β/a. The scanned images of tree-ring boundaries do not precisely match the standard superellipse curve shown in Figure 1. The existing programs of the ‘biogeom’ package in R software automatically estimate the coordinates of the pole in the plane and the angle of deviation of the major axis of the superellipse from the actual x-axis based on an optimization algorithm that minimizes the RSS (residual sum of squares) of the observed and predicted polar radii [35]. The superellipse equation assumes the bilateral symmetry of the tree-ring boundary. Although absolutely symmetric tree rings may not exist in nature. The predicted error between the scanned (observed) tree-ring boundary and the predicted tree-ring boundary by the superellipse equation for each sample was assessed using the adjusted root-mean-square error (RMSE_adj_). The ‘fitSuper’ function in the ‘biogeom’ package (version 1.3.5) [51] was performed to fit the superellipse equation within an R environment (version 4.3.1). The Nelder–Mead optimization method was used to estimate the parameters of superellipse equation to minimize the residual sum of squares (RSS) between the observed and predicted polar radii [52].

The area of a superellipse equation is calculated according to Strang and Herman [53]:

(3) $$\hat{A}=\int_{0}^{2\pi }\frac{1}{2}\;r^{2}d\varphi$$

where $\hat{A}$ is the predicted area, r and φ are the polar radius and polar angle, φ ∈ [0, 2π], and r of a superellipse equation is generated from Equation (2).

To assess the goodness of fit between observed and predicted tree-ring boundaries, the adjusted root-mean-square-error (RMSE_adj_) was calculated:

(4) $${RMSE}_{adj}=\frac{\sqrt{RSS/N}}{\sqrt{A/\pi }}$$

where A represents the observed area bounded inside each annual ring boundary, N represents the number of equidistant data points forming each tree-ring perimeter, and RSS is the residual sum of squares between predicted and observed polar radii [26,31].

The coefficient of variations (CV) of *k* and *n* among tree rings within each stem disc was calculated as follows:

(5) CV=σ/μ

where σ and μ are the standard deviation and mean of the dataset, respectively.

The analysis of variance (ANOVA) was used to test differences of CV and mean values of parameters *k* and *n* among different sites. To check the normality, a histogram and a normal quantile-quantile (Q-Q) plot were generated. The homogeneity of variances was assessed by plotting the residuals against the fitted values.

## 5. Conclusions

Our study verified that the shape of tree rings in *A. alba* trees tends to be bilaterally symmetric and that the superellipse equation can sufficiently describe the tree-ring shape of *A. alba*. The RMSE_adj_ ranged between 0.0038 and 0.0591, with a mean value of 0.0141. Soil moisture conditions influenced the size of the tree rings, not their shapes. The variation in tree-ring shape was more reflected in the inter-annual differences. Tree-ring shapes were hyperellipse (*n* > 2) in some years and changed to hypoellipse (*n* < 2) in other years. In addition, the sample tree with the largest *n*-CV exhibited a wide range of tree-ring shapes, i.e., ellipse, hypoellipse, hyperellipse, and even close to a rectangle-like with four rounded corners or a rhombus with four outwards curved corners. The four corners of the tree rings rotate as the tree ages, a phenomenon that is the cross-sectional representation of the trunk spiral grain. In future research, it would be interesting to compare spiral grain characteristics of different species using common garden experiments in conjunction with historical data on wind force and direction, while more research is needed to improve the validity and accuracy of methods for estimating forest productivity and carbon storage capacity.

## Figures and Tables

**Table 1:** Number of tree-ring samples from six different sites, the coefficients of variation (CV), and the mean values of parameters *k* and *n*.

Site	Tree NO	N ^1^	*k*-Mean	*k*-CV ^2^ (%)	*n*-Mean	*n*-CV ^2^ (%)
1008	1	37	0.970	2.057	2.039	4.154
1008	2	37	0.941	2.783	1.974	3.631
1008	3	40	0.961	3.184	2.011	2.592
1008	4	40	0.961	4.958	2.147	**12.026**
1008	5	38	0.950	1.707	1.975	4.999
1008	6	40	0.920	5.693	1.964	6.993
1009	7	37	0.957	1.567	2.008	3.471
1009	8	41	0.966	1.803	2.018	7.233
1009	9	30	0.951	4.355	2.043	6.680
1009	10	33	0.964	3.051	2.010	6.894
1009	11	36	0.917	4.156	2.078	8.394
1009	12	32	0.942	2.360	**1.933**	5.493
1010	13	41	0.973	1.925	2.002	4.833
1010	14	42	0.956	2.053	2.068	6.610
1010	15	36	**0.984**	2.967	2.096	5.792
1010	16	41	0.965	2.043	2.065	5.330
1013	17	40	0.944	3.895	2.092	8.132
1013	18	38	0.945	2.587	2.031	3.360
1013	19	42	0.935	2.494	1.969	5.937
1013	20	40	0.960	2.058	1.996	**2.488**
1013	21	40	0.917	**6.286**	2.063	3.129
1013	22	41	0.940	2.628	2.074	3.242
1014	23	42	**0.889**	5.333	**2.149**	6.663
1014	24	43	0.982	1.610	2.057	3.983
1014	25	41	0.962	4.939	2.005	7.141
1014	26	41	0.980	1.826	2.001	3.109
1014	27	41	0.980	2.794	2.027	3.380
1014	28	40	0.972	**0.983**	2.061	4.099
–	–	1090 ^3^	0.953 ^4^	3.003 ^4^	2.035 ^4^	5.350 ^4^

^1^ Number of tree rings; ^2^ *k*-CV, coefficient of variation of *k*. *n*-CV, coefficient of variation of *n*; ^3^ Total number of tree rings analyzed; ^4^ Average *k*-mean, *k*-CV, *n*-mean, and *n*-CV values, respectively.

**Table 2:** ANOVA data table for *k*-mean, *k*-CV, *n*-mean and *n*-CV.

Dependent Variables	Source	DF	Sum of Squares	Mean Square	F-Value	Pr (>F)
*k*-mean	Site	4	0.0025	0.0006	1.286	0.305
	residuals	23	0.0112	0.0005	-	-
*k*-CV	Site	4	3.970	0.993	0.462	0.763
	residuals	23	49.470	2.151	-	-
*n*-mean	Site	4	0.0075	0.0019	0.647	0.635
	residuals	23	0.0665	0.0029	-	-
*n*-CV	Site	4	15.290	3.822	0.774	0.553
	residuals	23	113.54	4.937	-	-

## Data Availability

Data will be made available upon request.
